# Molecular Mechanisms of Action of Tocotrienols in Cancer: Recent Trends and Advancements

**DOI:** 10.3390/ijms20030656

**Published:** 2019-02-02

**Authors:** Vaishali Aggarwal, Dharambir Kashyap, Katrin Sak, Hardeep Singh Tuli, Aklank Jain, Ashun Chaudhary, Vivek Kumar Garg, Gautam Sethi, Mukerrem Betul Yerer

**Affiliations:** 1 Department of Advanced Pediatric Center (APC), Postgraduate Institute of Medical Education and Research (PGIMER), Chandigarh, Punjab 160012, India; vaishali.pgi@gmail.com; 2 Department of Histopathology, Postgraduate Institute of Medical Education and Research (PGIMER), Chandigarh, Punjab 160012, India; make.must@gmail.com; 3 NGO Praeventio, 50407 Tartu, Estonia; katrin.sak.001@mail.ee; 4 Department of Biotechnology, Maharishi Markandeshwar (Deemed to be University), Mullana-Ambala 133207, Haryana, India; ashun.chaudhary@gmail.com; 5 Department of Animal Sciences, Central University of Punjab, City Campus, Mansa Road, Bathinda 151001, India; aklankjain@gmail.com; 6 Department of Biochemistry, Government Medical College and Hospital (GMCH), Chandigarh, Punjab 160031, India; garg.vivek85@gmail.com; 7 Department of Pharmacology, Yong Loo Lin School of Medicine, National University of Singapore, Singapore 117600, Singapore; 8 Department of Pharmacology, Faculty of Pharmacy, Kayseri 38039, Turkey; eczbetul@yahoo.com

**Keywords:** tocotrienols, cancer, apoptosis, anti-metastasis, anti-angiogenesis, miRNAs

## Abstract

Tocotrienols, found in several natural sources such as rice bran, annatto seeds, and palm oil have been reported to exert various beneficial health promoting properties especially against chronic diseases, including cancer. The incidence of cancer is rapidly increasing around the world not only because of continual aging and growth in global population, but also due to the adaptation of Western lifestyle behaviours, including intake of high fat diets and low physical activity. Tocotrienols can suppress the growth of different malignancies, including those of breast, lung, ovary, prostate, liver, brain, colon, myeloma, and pancreas. These findings, together with the reported safety profile of tocotrienols in healthy human volunteers, encourage further studies on the potential application of these compounds in cancer prevention and treatment. In the current article, detailed information about the potential molecular mechanisms of actions of tocotrienols in different cancer models has been presented and the possible effects of these vitamin E analogues on various important cancer hallmarks, i.e., cellular proliferation, apoptosis, angiogenesis, metastasis, and inflammation have been briefly analyzed.

## 1. Introduction

**Figure 1:**
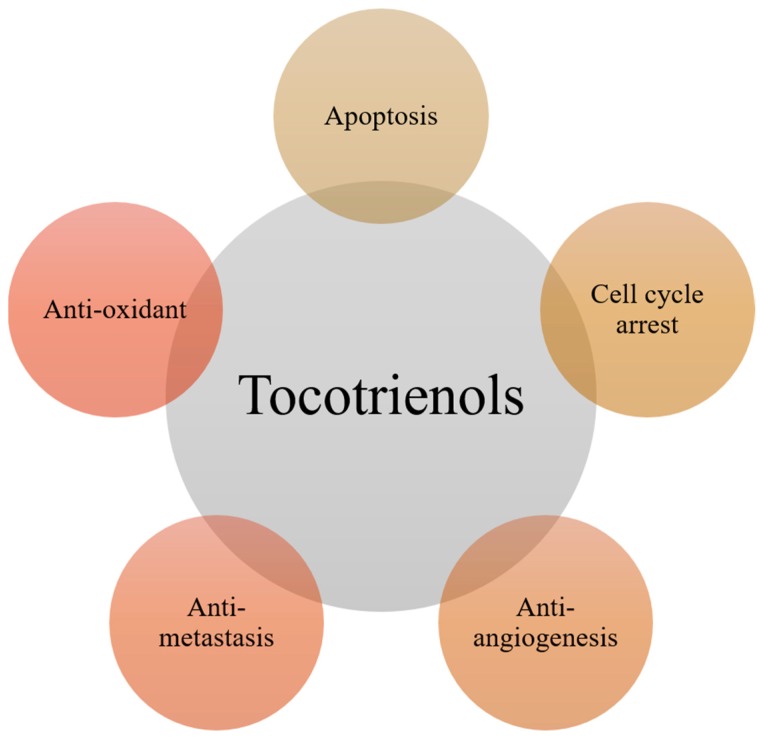
Anti-cancer properties of tocotrienols.

Cancer is a devastating and often fatal disease with an ever increasing incidence rate all over the world [1,2,3]. Based on recently published GLOBOCAN 2018 data, an estimated 18.1 million new cases of cancer and 9.6 million cancer deaths are expected in 2018 [4]. Therefore, research on molecular mechanisms of tumorigenesis, as well as the development of novel safe and effective anticancer agents are ever more important tasks for preclinical scientists, whereas transfer of the knowledge from bench to bedside must also be accelerated [5,6]. In the past decades, one of the major focus of anticancer research has been development of potent natural agents with cancer-combating properties [7,8,9]. Several natural compounds have been observed to exert antioxidant, antiproliferative, pro-apoptotic, anti-inflammatory, anti-angiogenic, anti-invasive, and anti-metastatic activities, thus acting as potential chemopreventive or chemotherapeutic agents [10,11,12,13,14,15,16,17,18,19,20,21,22,23,24,25,26]. One such group of compounds are tocotrienols, the unsaturated vitamin E analogues, which are found in several natural sources such as rice bran, annatto seeds and palm oil [27,28]. Tocotrienols can exist in different isoforms such as alpha (α), beta (β), gamma (γ), and delta (δ) (Table 1). Various beneficial properties of tocotrienols have been reported in recent years, especially against chronic diseases, including cholesterol-lowering, neuroprotective, antioxidant, anti-inflammatory, and anti-cancer properties [28,29,30,31,32,33]. Indeed, tocotrienols can suppress the growth of different malignancies, including those of ovary, liver, gastric, prostate, brain, blood, pancreas, and uterine [29,30] (Figure 1). In the current work, the reported anti-neoplastic effects of tocotrienols in different tumor models have been systematically reviewed.

## 2. Major Anti-Cancer Functions of Tocotrienols

### 2.1. Apoptosis Induction

Apoptosis is a critical process used as an innate defense mechanism against cancer initiation [34,35]. Several in vitro and in vivo experiments have confirmed the apoptosis inducing potential of natural molecules [36,37,38,39]. Tocotrienols are one among several natural compounds with effective antitumor activity via apoptosis inducing pathways [40]. Researchers conducted a study in 1999 and concluded that tocotrienols (α, γ and δ) and RRR-δ-tocopherol induced substantial apoptosis in breast cancer cell lines except the tocopherols (α, β, and γ) and the acetate derivative of RRR-α-tocopherol (RRR-α-tocopheryl acetate) [41]. Ahn and coworkers demonstrated that γ-tocotrienol (γ-T3) down-regulated the expression of several oncogenic gene products by inhibiting NF-κB pathway and also caused substantial apoptosis in tumor cells [42]. Further, Yap et al., in 2008 suggested that treatment of PCa cells with γ-T3 induced growth inhibitory effects by affecting several signaling pathways and showed chemosensitization and anti-invasive effects of γ-T3 against prostate cancer cells [43]. Another study suggested that Bcl-2 family members and caspase-3 were the key regulators in γ-T3-induced apoptosis in gastric cancer SGC-7901 cells and apoptosis was mediated through the down-regulation of the Raf-ERK signaling pathway [44]. 

Narimah et al., demonstrated that γ-T3 and α-tocopherol showed anti-proliferative activities and induced apoptosis in Caski (cervical carcinoma cell line) and Alexander cells (hepatoma cell line) [45]. It has also been found that α-T3 and γ-T3 were more efficient than δ-T3 and α-tocopherol in inhibiting proliferation in human cervical cancer HeLa cells by up-regulating IL-6 and down-regulating cyclin D3, p16, and CDK6 expression [46]. Similarly, another report suggested that tocotrienols can induce apoptosis in breast cancer cell lines via upregulation of DR5 that was dependent on JNK and p38 MAPK activation and mediated through endoplasmic reticulum stress [47]. Selvaduray et al., in 2010 reported that tocotrienols can exert significant antiangiogenic activity and induce apoptotic effects associated with increased levels of IL-24 mRNA in BALB/c mice [48]. The authors revealed that γ-T3 can alter endoplasmic reticulum stress signaling and identified the activating transcription factor 3 as possible molecular target for γ-T3 in breast cancer cells (MDA-MB 231 and MCF-7) [49]. In few another reports, the authors concluded that tocotrienols induced apoptosis and anti-proliferative activity in two human breast cancer cell lines, triple negative breast cancer (TNBC) cells (MDA-MB-231) [50] and oestrogen-dependent cells (MCF-7) by causing DNA fragmentation, NF-κB inhibition, and poly (ADP-ribose) polymerase cleavage [51]. 

**Figure 2:**
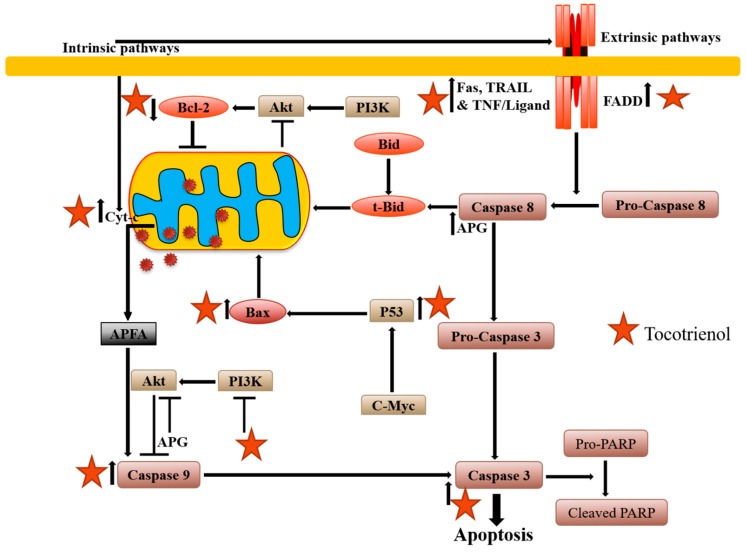
Diagrammatic representation of tocotrienols-mediated apoptosis activation. Tocotrienols can regulate both intrinsic and extrinsic apoptotic pathways via mitochondrial membrane potential depolarization, cyt. C (cytochrome complex) release, activation of caspase cascade and also modulation of Bcl-2 (B-cell lymphoma 2) as well as Bax expression.

In another study, the authors showed that δ-T3 has higher efficacy and effectiveness in carrying out apoptosis in both human lung adenocarcinoma A549 and glioblastoma U87MG cells as compared to α- and γ-T3 [52]. In 2015, Wang et al., treated MDA-MB-231 cells with δ-T3 and found that the latter effectively induced apoptosis in these cells by activating miR-429 [53]. Moreover, it was noted that δ-T3 arrested the growth of human bladder cancer cells, induced apoptosis, and chemosensitization by inhibiting STAT3 pathway [54]. Lovastatin and γ-T3 induced differentiation and apoptosis in HL-60 cells via Ras/Akt/NF-κB and Ras/ERK/NF-κB signaling dependent pathways by down-regulating glyoxalase 1 and HMG-CoA reductase enzymes [55]. Another report also found that γ-T3-induced ER stress-mediated apoptosis and autophagy in breast cancer MCF-7 and MDA-MB-231 cells [56]. γ-T3 acted as an effective inducer of apoptosis in K562 chronic myeloid leukemia cells, which was mediated by both extrinsic and intrinsic apoptotic pathways [57]. Rajasinghe and Gupta showed that tocotrienol-rich mixture hindered cell proliferation, migration, and tumor cell invasiveness by downregulating NF-κB and Notch-1 pathways during apoptosis induction in non-small cell lung cancer cells (NSCLC) [58]. In 2017, Xu et al., concluded that γ-T3 acted as anti-proliferative agent and induced apoptosis in HeLa cells via the mitochondrial pathway [59]. Overall, tocotrienols can exert their apoptotic effects through diverse mechanism(s), which are briefly summarized in Figure 2.

### 2.2. Cell Cycle Arrest 

**Figure 3:**
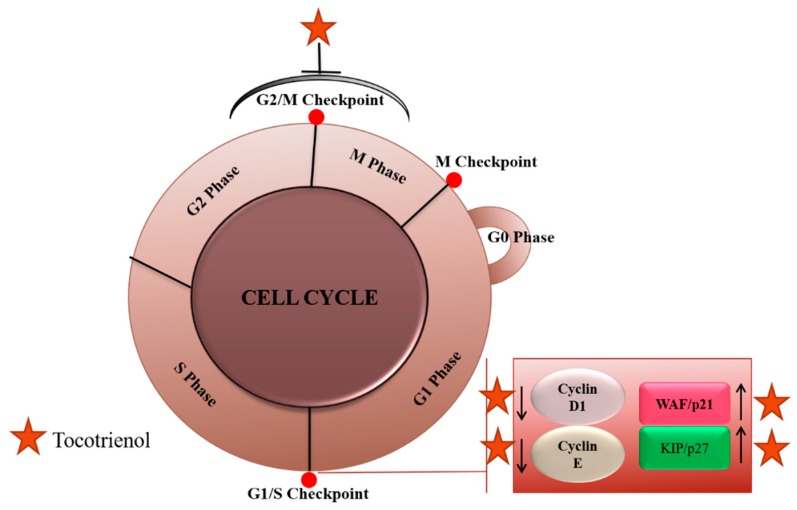
Tocotrienols mediated inhibition of proliferation through their effects on cell cycle regulation.

Cell cycle checkpoints, also considered as a therapeutically targetable double-edged sword, elicit cell cycle safeguard mechanisms [60]. Aberrant activation of cell cycle proteins manifests into uncontrolled tumor cell proliferation. Hence, targeting cell cycle checkpoints can be an important strategy for cancer therapy [61]. Recent scientific evidence has documented the potential role of tocotrienols as therapeutic agents in cancer treatment through the modulation of cell cycle proteins in pancreatic cancer [62,63], leukemia [64,65], glioblastoma [66], gastric cancer [67], neuroblastoma [68] etc. In another study, Abubakar et al., (2017) documented the anti-proliferative potency of γ-T3 and jerantinine A in brain cancer (U87MG) cells, and found that the combination can lead to G0/G1 cell cycle arrest and trigger disruption of microtubule networks, thus promoting Fas and p53 induced apoptosis mediated through the death receptor and mitochondrial pathways (Figure 3) [69]. 

In an interesting study carried out in MCF-7/Adr breast cancer cells, γ-T3 was reported to reverse multi-drug resistance through inhibition of P-gp expression and increased cellular accumulation of doxorubicin, which led to elevated G2/M arrest and apoptosis in breast cancer cells [70]. Sato and colleagues (2017) deciphered the combination effect of γ-tocopherol and δ-T3 in human androgen-dependent prostate cancer cells (LNCaP), leading to simultaneous cell cycle arrest in the G1 phase and G2/M phase significantly inhibiting prostate cancer cell growth [71]. Yeganehjoo et al., reported in 2017 that the synergistic impact of d-δ-T3 and geranyl geraniol induced concentration dependent suppression of human DU145 prostate carcinoma cells via cell cycle arrest in G1 phase acting through down-regulation of 3-hydroxy-3-methylglutaryl coenzyme A (HMG CoA) reductase of mevalonate pathway and K-ras [72]. γ-T3 was also reported to arrest cell cycle at G0/G1 phase and reduce the S phase in HeLa cells [59]. 

This perspective discussion on the role of tocotrienols in the induction of cell cycle arrest based on recent scientific observations will indeed have a broader impact on the treatment of human cancers in the near future. Hence, the administration of tocotrienols in combination with chemotherapeutic agents can show significant growth inhibitory effects through check-point regulators. The promising scientific evidence(s) related to the bioactive potential of tocotrienols supports their pharmacological development as novel clinical approaches in checkpoint modulation.

### 2.3. Angiogenesis Inhibition Potential of Tocotrienols

Angiogenesis, defined as the formation of new blood vessels from preexisting vasculature, is an essential process for tumor cell proliferation and viability [73,74]. The complexity of angiogenesis is entangled in its regulation through numerous pro- and anti-angiogenic factors. Targeting these angiogenic factors has long been explored extensively on the therapeutic front for improving prognosis in cancer; however, with limited efficacy so far [75,76]. The pre-clinical research has emphasized the promising anti-angiogenic/anti-neoplastic effects of tocotrienols in various cancers [26,77,78,79,80], the findings of which are briefly summarized below.

**Figure 4:**
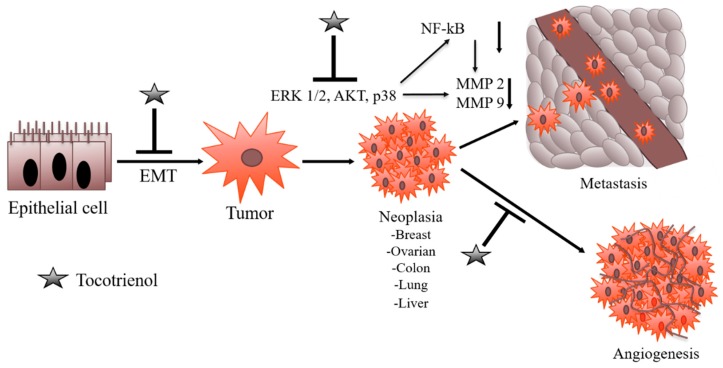
Tocotrienols also mitigate tumor metastasis and angiogenesis by regulating the MAPK (mitogen-activated protein kinases), Akt signaling pathways and subsequently MMPs (matrix metalloproteinase), snail, twist, β-catenin, and uPA gene expression. Tocotrienols also reduce the activity/expression of HIF-1α (hypoxia-inducible factor 1-alpha) and VEGF/VEGFR (Vascular Endothelial Growth Factor and Its Receptor) molecules.

Yang et al., (2007) suggested the possible involvement of Raf/MEK/ERK signaling cascade in angiogenesis [81]. δ and γ-T3 were also reported to suppress Ras-Raf-MEK-ERK pathway-associated upstream signaling, which in turn can inhibit angiogenesis and proliferation in human hepatocellular carcinoma (HepG2) cells [82]. Rajasinghe and colleagues in 2017 reported the bioactive potential of tocotrienol rich mixture in inhibiting proliferation in lung adenocarcinoma (A549) and squamous cell lung cancer (H520) cell lines via downregulation of NF-κB and downstream pro-angiogenic molecules (Figure 4) [58]. In another study carried out by Husain and colleagues, it was demonstrated that δ-T3 inhibited biomarkers of tumor angiogenesis (VEGF and MMP-9) in pancreatic cancer cells (L3.6pl and MiaPaCa-2) in vitro and decreased the expression of CSCs cell surface markers (CD31 and CD44) [83]. Also, δ-T3 has exhibited significant efficacy against both melanoma and its associated stem cells [84,85].

Kaneko et al. (2018) have also clearly demonstrated the suppressive actions of δ-T3 on hypoxia adaptation of prostate cancer stem-like cells [86]. Shiozawa et al., have also elegantly illustrated the cytotoxic effect of redox-silent analogue of tocotrienol in inhibiting survival of PC3 cells (androgen-independent prostate cancer (PCa) cell line) under hypoxia, through inhibition of Fyn/HIF-1α signaling cascade, which may lead to the establishment of a new effective therapy for PCa [87]. In addition, pre-clinical data from numerous other studies have also reported that γ-T3 alleviates angiogenic protein expression (VEGF) in colorectal cancer [88], malignant mesothelioma [89], breast cancer [90], ovarian carcinoma [91], head and neck squamous cell carcinomas [92], and other cancers. Thus, in view of the above scientific reports, tocotrienols appear to be important therapeutic molecules for improvement of prognosis in cancer patients by targeting angiogenesis. 

### 2.4. Suppression of Metastasis

Tumor metastasis, a dynamic process, is regulated by numerous intrinsic and extrinsic mechanisms which dictate the molecular and cellular basis of metastatic cancer progression rendering cancers refractory to therapeutic treatment [93]. Scientists are carrying out extensive research to identify the therapeutic compounds and treatment strategies to target cancer metastasis. Tocotrienols have gained scientific prominence recently due to their promising anti-cancer and anti-metastatic properties. The signaling pathways that regulate extracellular matrix and tumor cell motility during tumor invasion have been reported to be modulated by tocotrienols [94]. Rajasinghe and colleagues in 2018, documented that δ-T3 can alleviate tumor invasion and metastasis in A549 and H1299 cells by repression of protease activity of MMP-9/urokinase-type plasminogen activator (uPA) through down-regulation of Notch-1 and NF-κB pathways and up-regulation of miR-451 in NSCLC (Figure 4) [95]. 

Interestingly, Husain et al., also documented the evidence of the bioactive potential of δ-T3 to inhibit pancreatic ductal adenocarcinoma cancer (PDAC) stem-like cells and prevent pancreatic cancer metastasis. This study further reported the inhibitory effect δ-T3 on processes underlying metastasis such as migration, invasion, and epithelial-to-mesenchymal transition (E-cadherin, N-cadherin, and Vimentin) in PDAC cells and tumors. The in vitro findings from this study corroborated with the in vivo results in orthotopic xenograft mouse models (*LSL-KRAS^G12D^/PDX-1-Cre* and *LSL-Kras^G12D/+^; LSL-Trp53^R172H/+^; Pdx-1-Cre* [KPC]) via down-regulation of Oct4 and Sox2 transcription factors [83]. In human colorectal cancer cell lines (HCT-116, HT-29 and Caco-2), γ-T3 administration in conjunction with capecitabine was reported to attenuate proteins associated with survival (Survivin, cIAP-1, and cIAP-2), proliferation (cyclin D1 and c-myc), and metastasis (MMP-9, VEGF, ICAM-1, and CXCR4) by down-regulating NF-κB/p65 pathway [88]. 

An interesting study published in 2017 reported that tocotrienols inhibited the growth of prostate cancer through epigenetic modifications leading to elevation of H3K9 acetylation levels which induced cyclin-dependent kinase inhibitors p21 and p27 [96]. Xu et al. (2017) deciphered that γ-T3 acted through mitochondrial pathway (down-regulation of Bcl-2, up-regulation of Bax, the release of mitochondrial cytochromes, activation of caspase-9, and caspase-3 leading to poly (ADP-ribose) polymerase (PARP) cleavage) in HeLa cells [59]. Several studies have documented significant therapeutic and anti-metastatic potential of tocotrienols in breast cancer as well. Ahmed and coworkers in 2016 concluded that reversal of epithelial-mesenchymal transition by γ-T3 in MDA-MB-231 and T47D cells was associated with the attenuation of canonical Wnt signaling [97]. 

Another study demonstrated that γ-T3 treatment significantly reduced metastatic phenotypic expression in highly metastatic mouse +SA and human MDA-MB-231 breast cancer cells through dose-dependent down-regulation of Rac1/WAVE2/Arp2/3 signaling that plays an important role in cytoskeletal reorganization and formation of membrane protrusions [98]. The investigational results from another study reported that γ-T3 alone or in combination with docosahexaenoic acid (DHA) eliminated ALDH+ human TNBC, inhibited mammosphere formation through the upregulation of Src homology region 2 domain-containing protein tyrosine phosphatase-1 (SHP-1) and suppression of STAT3 signaling, along with its downstream mediators c-Myc and cyclin D1 [99]. Besides the above-mentioned literature, a plethora of studies have emphasized the anticancer/anti-metastatic potential of tocotrienols in colon cancer [100,101], pancreatic cancer [62,102], breast cancer [103], ovarian cancer [91], melanoma [104], and neuroblastoma [105]. The findings from quoted literature suggest the promising safety profile of tocotrienols in healthy subjects, along with anti-tumor effects which suggest that the administration of tocotrienols should be ardently explored on the therapeutic front [30].

### 2.5. Regulation of Non-Coding RNAs

In the previous sections, we described various ways in which tocotrienols can modulate a variety of cellular functions and exert therapeutic efficacy against malignancies. However, the mechanism(s) behind the effect of tocotrienols in influencing the expression of non-coding RNAs (ncRNAs) is still not very clear. Recent advancement in whole genomic and transcriptomic analysis has revealed that more than 90% of the human genome is transcribed as regulatory non-coding RNAs (ncRNAs), such as Piwi-interacting RNAs (piRNAs), small-interfering RNAs (siRNAs), circular RNAs (circRNAs), small Cajal body-specific RNAs (scaRNAs), small nucleolar RNAs (snoRNAs), microRNAs (miRNAs), and long non-coding RNAs (lncRNAs) [106,107,108,109,110,111]. The above-mentioned ncRNAs are found to play important roles in regulating the various biological processes, and alterations in their expression may have drastic effects on cellular functions and can contribute to disease etiology, including cancer [112,113,114]. miRNAs can play an important role in cancer by regulating the expression of various oncogenes and tumor suppressor genes through targeting the 3′-untranslated region of mRNA [115,116,117]. As we only found literature relevant to the effects of δ-T3 on the regulation of miRNAs, we next describe how these tocotrienols may alter the expression and function of miRNAs.

In this context, Wang C, 2015 et al. [53] evaluated the effects of δ-T3 on MDA-MB-231 cells. The authors found that the treatment with δ-T3 inhibited the proliferation of these cells in a dose-dependent manner. To explore the involvement of miR-429 in δ-T3-induced apoptosis in MDA-MB-231 cells, the authors performed real-time PCR and caspase-3 activity assays. Interestingly, they found that δ-T3-treated cells significantly expressed a low level of miR-429 compared to the untreated cells. In addition, the δ-T3 treatment resulted in a marked induction of caspase-3 activity and apoptosis in the cells transfected with control miRNAs compared to those transfected with the miR-429 inhibitor. Furthermore, they observed that inhibition of miR-429 partially rescued the apoptosis induced by δ-T3 in MDA-MB-231 cells. Moreover, X-linked inhibitor of apoptosis protein (XIAP) was identified as one of the target genes regulated by miR-429.

The miR-429 has been reported to be significantly down-regulated in several cancers, including renal cell carcinoma [118] and gastric cancer. Emerging evidence has shown that over-expression of miR-429 can inhibit proliferation and induce apoptosis in human osteosarcoma cancer cell lines [119]. Similarly, Ji et al., 2012, studied the effect of δ-T3 on the global miRNA expression in NSCLC. By miRNA array analysis, the authors found that δ-T3 significantly up-regulated the expression of miR-34a in treated H1650 and A549 cell lines compared to the untreated cells. They also observed a significant increase in miR-34a expression in the δ-T3-treated cells compared to control in a dose and time-dependent manner. The relative expression of miR-34a after 72 h were found to be ~118- and ~120-fold higher than the control in A549 and H1650 cell lines, respectively. Furthermore, it was observed that cell proliferation was inhibited by 74% upon δ-T3 exposure alone in A549 cells, whereas the combination treatment of AS-miR-34a (miRNA-34 inhibitor) and δ-T3 reduced it by 57%. Mechanistically, it was demonstrated that δ-T3 inhibited the cell proliferation and invasion by up-regulating the expression of miR-34a and suppressing Notch-1 signaling pathway [120]. 

### 2.6. Role as Antioxidants

**Figure 5:**
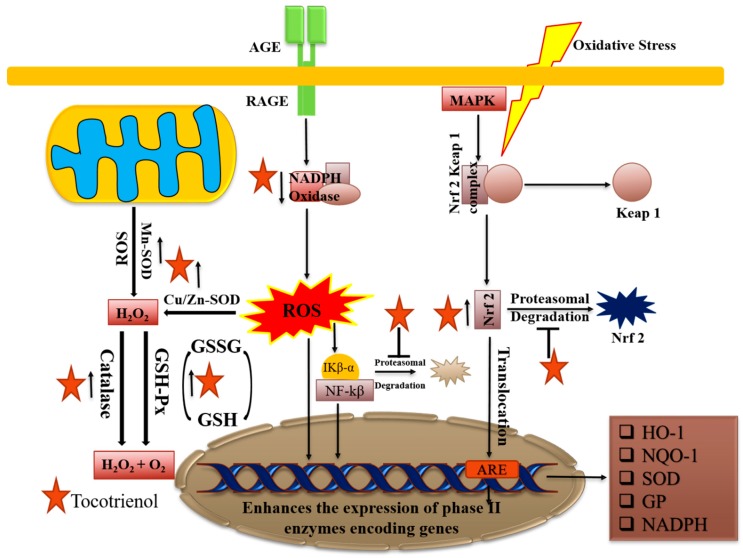
Tocotrienols can act against oxidative stress and increase the expression of anti-oxidant enzymes such as GSH (glutathione)-synthase, catalase, and SOD (superoxide dismutase). They can block the NADPH ((nicotinamide adenine dinucleotide phosphate) oxidase complex and their downstream target inflammatory genes and increase the nuclear translocation of Nrf-2 (nuclear factor erythroid 2–related factor 2).

Antioxidants have also been explored for their anticancer properties, and there are mainly two types: direct and indirect. Direct antioxidants are substances that can inactivate free radicals or prevent free-radicals-initiated chemical reactions, e.g., glutathione, tocotrienols, ascorbic acid, and carotenoids. Indirect antioxidants are not able to participate in redox reactions, but they enhance the antioxidant ability of cells by several mechanisms and hence provide protection against oxidative stress. The oxidative state is maintained with complex systems of overlapping antioxidants, producing internal and dietary antioxidants like vitamin C and vitamin E. The secondary plant metabolites in fruits and vegetables, such as tocotrienols, carotenoids, phenolic acids, flavonoids, etc., which inhibit oxidation reaction, are called antioxidants [28,121]. These compounds intervene in cellular defense mechanisms that can produce free radicals or cellular metabolism, thereby leading to termination of chain reactions that may damage cells of the organisms. In healthy cells, the usual oxidants are formed in an inhibited way and can regulate several important physiological functions. The in vitro and in vivo literature findings from the last few decades reveal that tocotrienols have antioxidant, neuroprotective, and cholesterol lowering properties, thereby exerting various health beneficial effects [122,123]. The evidence also suggested that the mechanism of tocotrienols included selective control of the Nrf-2-Keap1 coordination by altering the expression of antioxidant modulatory enzymes (Figure 5) [124]. 

### 2.7. Anti-Inflammatory Effects

Accumulated findings have established that tocotrienols as an anticancer agent were primarily mediated by affecting multiple oncogenic cascades [43,51,54,121,125], including the inhibition of two important anti-inflammatory transcription factors, namely, NF-κB and STAT3 [30,32,33,121] as well as their downstream gene products involved in proliferation and survival in several cancer cell lines [30,31,32,42,43,51,88,126]. Moreover, δ-T3, inhibited Src kinase, Janus kinase (JAK) 1, and JAK2, thus eventually downregulating STAT3 activation in bladder cancer cells [54]. Interestingly, γ-T3, was found to abrogate STAT3/5 and Akt activation in combination with erlotinib/geftinib in murine mammary tumor cells [127]. It has also been reported that tocotrienols can inhibit cyclooxygenase-2 (COX-2)-mediated production of prostaglandin E2 (PGE2), in IL-1β treated A549 cells and γ-T3 was found to be one of the most potent vitamin E isoforms in this study [128]. Moreover, Shibata et al., showed that HIF-1α regulated production of angiogenic molecules can also be attenuated by tocotrienols [129].

## 3. Selected In Vivo Studies

Due to a close resemblance with the human diseases, in vivo model systems are commonly used to understand disease etiology. Numerous anti-cancer effects of tocotrienols as well as their active derivatives in various preclinical models have been briefly summarized in Table 2.

## 4. Conclusions and Future Perspective

A wide variety of bioactive compounds isolated from the natural products [136,137,138,139,140,141,142,143,144,145,146], including tocotrienols, possess a vast range of therapeutic benefits and might be useful for both the prevention and treatment of cancer. The mechanisms of action of tocotrienols at the cellular and molecular level suggest the modulation of various cancer-related signaling pathways. However, more experimental evidence is required to further explore the documented biological functions of tocotrienols by using sophisticated approaches such as nanotechnology and quantitative structure-activity relationship models. It may also be unraveled using docking-based software employed for new drug discovery and development. It appears that in the future, well-designed clinical trials with tocotrienols would lend additional support to the findings of previous investigators and lead to the development of tocotrienols as cost-effective therapeutic agents for treating different types of malignancies.

## Figures and Tables

**Table 1:** Chemical structures of different isoforms of tocotrienols.

Isoform Name	Chemical Structure
Alpha(α)-Tocotrienol	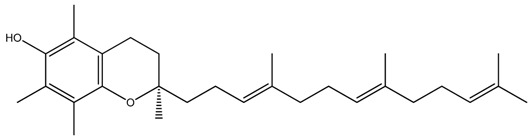
Beta(β)-Tocotrienol	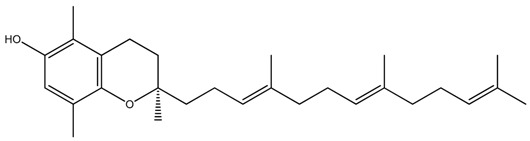
Gamma(γ)-Tocotrienol	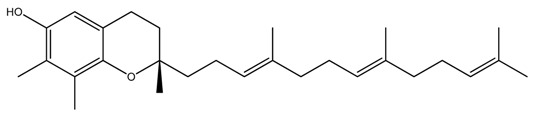
Delta(δ)-Tocotrienol	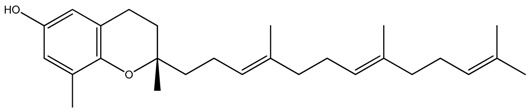

**Table 2:** A brief overview of in-vivo studies carried out using tocotrienols.

Mechanisms	Model Systems	Dose	Ref.
- Reduces activation of AKT, NF-κB - Mitigates the levels of COX2, cyclin D1, CDK2, CDK4, and CDK6 - Elevates the expression of p21 and p27	Mammary syngeneic model	2–5 µM	[130]
- Modulates cell cycle regulatory proteins - Increases expression of pro-apoptotic proteins	TRAMP mouse model	0.3% and 1%	[131]
- Attenuates tumor growth	Mammary syngeneic model	0.5 mg/day	[132]
- Reduces expression of Ki-67, COX-2, MMP-9, NF-κB p65, VEGF - Down regulates expression of cyclin D1, c-myc, VEGF, and CXCR4	Orthotopic pancreatic cancer	400 mg/kg	[126]
- Reduces increased neovascularization	Angiogenic models	10 mg/day	[133]
- Increases apoptosis - Increases senescence-like growth arrest	Mammary HER-2/neu transgenic mouse model	50 or 100 mg	[134]
- Reduces Ki-67, cyclin D1, MMP-9, CXCR4, NF-κB/p65, and VEGF	Xenograft colorectal cancer model	-	[88]
- Inhibits DLD-1-induced vessel formation	Mouse matrigel plug assay	0–20 µg	[135]
- Suppresses activation of AKT/mTOR pathway - Inhibits vessel formation, tumor growth and angiogenesis	Orthotopic liver cancer	3.25 mg	[31]
- Affects the activity of anti-oxidative enzymes and Wnt pathway - Inhibits tumor growth	Xenograft colon cancer model	5, 10 and 20 mg/kg	[125]
